# Full-Tree Biomass, Root Carbon Stock, and Nutrient Use Efficiency Across Ages in *Eucalyptus* Stands Under Seedling and Coppice Systems

**DOI:** 10.3390/plants14091382

**Published:** 2025-05-03

**Authors:** Gardenia Gonçalves de Oliveira, Túlio Barroso Queiroz, Bronson P. Bullock, José Carlos Coelho, Rodrigo Eiji Hakamada, Iraê Amaral Guerrini

**Affiliations:** 1 Department of Forest, Soil and Environmental Sciences, College of Agricultural Sciences, São Paulo State University-UNESP, Botucatu 18610-034, SP, Brazil; tulioqueiroz@uga.edu (T.B.Q.); jose.coelho@unesp.br (J.C.C.); irae.guerrini@unesp.br (I.A.G.); 2 Center for Carbon Research in Tropical Agriculture (CCARBON), University of São Paulo, Piracicaba 13418-900, SP, Brazil; rodrigohakamada@usp.br; 3 Plantation Management Research Cooperative (PMRC), Warnell School of Forestry & Natural Resources, University of Georgia, Athens, GA 30602, USA; bronsonbullock@uga.edu; 4 Forest Science Department, University of São Paulo, Piracicaba 13418-900, SP, Brazil

**Keywords:** biomass production, biological utilization coefficient, forestry nutrition

## Abstract

The establishment of forest stands after harvest requires an understanding of biomass and nutrient dynamics to support management decisions and ensure system productivity and sustainability. This study evaluated biomass and nutrient accumulation in *Eucalyptus urophylla* aged 2 to 5 years under planting and coppicing systems. A total of 1152 trees were assessed across eight treatments, combining four ages and two management systems. Aboveground biomass was estimated using 10 trees per treatment, while root biomass was assessed in 8 trees at ages 3 and 5. Nutrient concentrations were determined using three intermediate-diameter class trees per treatment. Biomass data were analyzed using Tukey’s test (5%), and biomass expansion factors (BEF) and the root-to-shoot ratio (R) were used to estimate root carbon. Total biomass was higher in the coppicing system (153 Mg ha^−1^) compared to the planting system (119 Mg ha^−1^), with greater root accumulation and carbon sequestration (≈17.2 t C ha^−1^). The biological use coefficient (BUC) increased with age, except for Mn. Planted stands showed higher BUC for N and P, while coppiced stands were more efficient in Mg use. These results reinforce the need for distinct fertilization strategies for each system, aiming at productivity, nutrient efficiency, and carbon stock enhancement.

## 1. Introduction

With growing prominence in economic and environmental agendas, the genus *Eucalyptus* has established itself as one of the pillars of global forestry, supporting productive chains and sustainable land use strategies in several countries. Its versatility, adaptability to different edaphoclimatic conditions, and wide range of industrial applications make it one of the most widely cultivated species in forest plantations. According to the 2020 Global Forest Resources Assessment (FRA), published by the Food and Agriculture Organization of the United Nations [1], *Eucalyptus* plantations occupy approximately 19 million hectares worldwide, reinforcing their strategic role in contemporary forest systems. In addition to its high growth rate, quality wood, and ability to meet various industrial demands, the genus is widely recognized for its remarkable ability to resprout after harvesting, a characteristic that expands management possibilities and enables sustainable alternatives such as the coppicing system. This system relies on forest regeneration through sprouts that emerge from the stumps after clear-cutting, eliminating the need for replanting seedlings and making use of the already established root system [2,3,4,5].

The comparison between coppice systems and seedling-based plantations is essential for the development of sustainable forestry practices, as it allows for the assessment of the balance between productivity, conservation of natural resources, and economic viability. Coppicing, by reusing the pre-existing root system, enables faster regeneration with lower demand for soil preparation and inputs, resulting in reduced environmental impact and lower operational costs [4,6,7]. However, its performance may be affected by factors such as nutrient depletion, damage to stumps during clear-cutting, limited resprouting capacity in successive rotations, and unfavorable water regimes [6,8,9]. On the other hand, planting with seedlings offers greater control over the initial establishment of trees but involves higher costs and greater soil disturbance [10,11]. Therefore, comparing both systems under different edaphoclimatic and genetic conditions provides a better understanding of their limitations and potential, guiding management decisions that promote the long-term sustainability of forest production [12,13]. Given the similar availability of water, light, nutrients, oxygen, and temperature, biomass production in coppice stands tends to be comparable to that of the first rotation of cultivation [12,13]. However, potential reductions in productivity in this system may be related to factors such as lower nutrient availability [8,12], stump damage during harvesting [9], low sprouting capacity, unfavorable water regimes [6], and intensive soil disturbance caused by inadequate operational practices.

In light of these potential challenges, it becomes evident that the success of the coppice system is intrinsically linked to the adoption of efficient and well-guided forest management. Achieving high productivity in forest stands requires not only the appropriate selection of species and clones but also practices that preserve the site’s productive capacity over rotations [10]. Understanding the growth of *Eucalyptus* throughout its phases is, therefore, essential for grounding more sustainable management decisions. In the early stage of canopy closure, trees grow vertically and expand their crowns in a less competitive environment, promoting accelerated growth [11]. As the stand develops, competition for resources such as sunlight, water, nutrients, and space intensifies, which can lead to morphological and physiological changes in the trees [4,14,15].

Understanding nutrient demand that is, the quantity and type of essential elements that trees need to maintain their growth, development, and productivity over time is crucial for guiding more efficient forest management practices in plantations. Simultaneously, the study of carbon stocks in *Eucalyptus* forests has gained prominence, especially in sustainable management contexts, where the goal is to combine productivity with environmental conservation. In systems managed both by planted seedlings and by coppicing, the dynamics of nutrients and carbon allocation, particularly in coarse roots, are critical aspects for maintaining soil fertility and the longevity of the stands. These roots perform vital functions in the accumulation of belowground biomass, acting as important long-term carbon reservoirs and significantly contributing to carbon sequestration. Furthermore, they directly influence nutrient absorption, transport, and redistribution in the plant-soil system, which in turn affects tree growth [4,16]. Therefore, investigating nutrient demand and carbon stocks in coarse roots provides essential insights for improving silvicultural practices, promoting a balance between productivity and sustainability, and contributing to more effective climate change mitigation strategies.

Considering the critical importance of biomass and nutrient allocation for sustainable forest management, this study aims to quantify biomass, nutrient stocks, and carbon in the coarse roots (diameter > 2 mm) of *Eucalyptus urophylla* under two distinct management systems: coppicing and seedling planting. The assessment, conducted at ages ranging from two to five years post-harvest or planting, provides insights into the temporal dynamics of these compartments. Additionally, the study seeks to estimate the nutritional demand of the wood through the biological utilization coefficient (BUC). We hypothesize that the coppice system may achieve nutrient efficiency and carbon storage levels comparable to, or even exceeding, those of the seedling based system, owing to the regeneration of pre-existing roots and enhanced stability in coarse root carbon stocks. The importance of process based models is highlighted in this context, as they offer a powerful framework for understanding the ecological and physiological interactions within forest ecosystems, allowing for more precise predictions of how different management practices influence nutrient cycling, carbon sequestration, and overall ecosystem functioning.

## 2. Results

### 2.1. Biomass Stock

**Figure 1:**
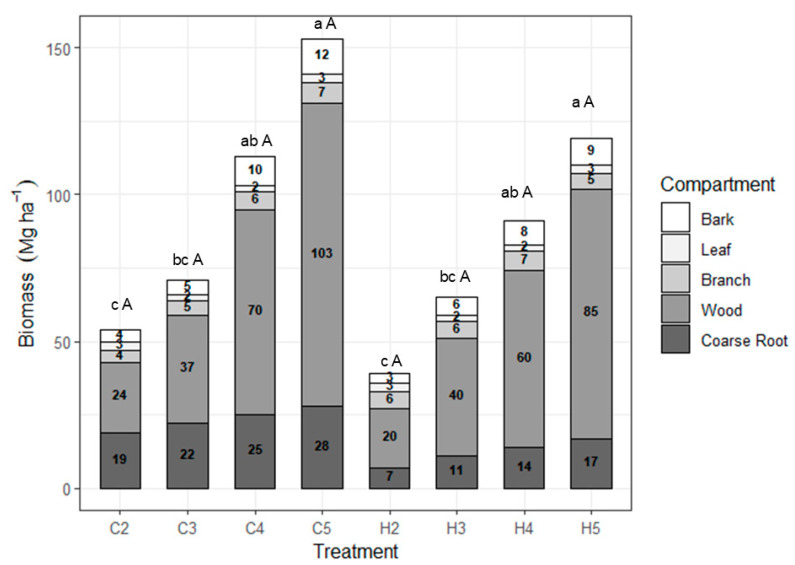
Biomass accumulation (Mg ha^−1^) of *Eucalyptus urophylla* from two to five years of age under coppice management and seedling system. Where: C2 = Coppice at 2 years, C3 = Coppice at 3 years, C4 = Coppice at 4 years, and C5 = Coppice at 5 years; S2 = Seedlings at 2 years, S3 = Seedlings at 3 years, S4 = Seedlings at 4 years, and S5 = Seedlings at 5 years. Means followed by the same letter do not differ significantly from each other at the 5% probability level according to Tukey’s test. Uppercase letters compare management systems within each age, while lowercase letters compare ages within each management system.

The coppice management tended to have higher total biomass accumulation at all ages (Figure 1). Despite soil peculiarities among treatments, there were no significant differences in total biomass growth between establishment seedlings and coppicing systems of the same age.

Age was the main factor directly influencing bark, wood, and coarse roots biomass; on the other hand, leaves and branches showed no significant variation in biomass accumulation with increasing age. Coarse root biomass was the only compartment that showed a significant difference, particularly in coppice management, where it demonstrated greater growth (165 to 271%) across all ages.

**Figure 2:**
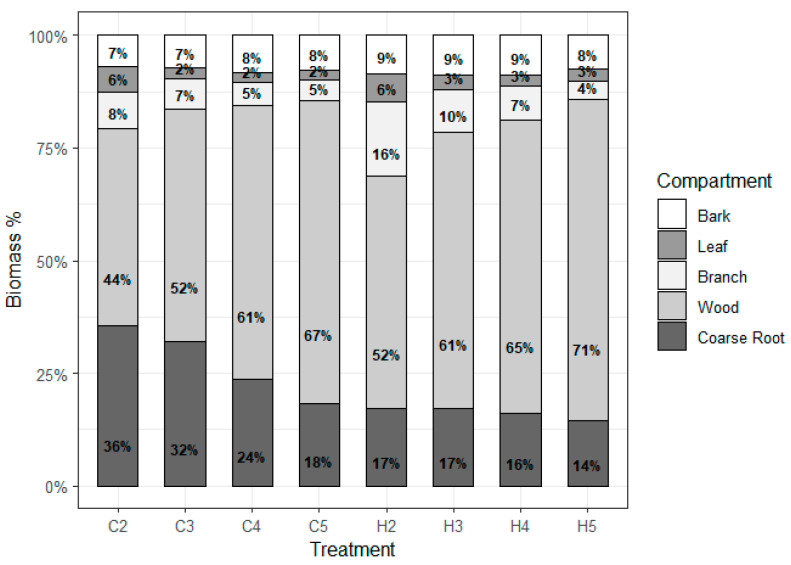
Percentage biomass distribution of *Eucalyptus urophylla* by tree compartment (bark, leaves, branch, wood, and roots) at 2, 3, 4, and 5 years of age, under coppice and seedling planting systems. The bars represent the relative biomass allocation to each tree component at different ages and management systems.

The relative distribution of biomass in different components follows the following order at 2 and 3 years in both management systems: Wood > Root > Branch > Bark > Leaf (Figure 2). After the fourth year, there is a reversal in the order (bark and branch), which becomes the following: Wood > Root > Bark > Branch > Leaf.

After the wood component, the second component that shows higher production at all ages and managements is the roots. Regarding the change in components over time, except for the wood component, which increased, and the bark, which maintained a constancy in relation to its contribution to the total biomass in all treatments, there was a reduction in the other components (roots, branches, and leaves) as age increased.

### 2.2. Nutritional Demand

The BUC showed lower utilization efficiency at two years of age, due to the greater initial investment that trees make in the absorption of these elements. Consequently, the BUC increased with age, except for Mn, becoming more efficient as age advanced for most nutrients.

**Figure 3:**
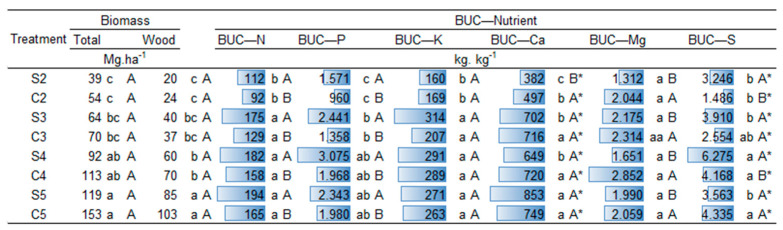
Biological utilization coefficient (BUC—nutrient units per unit of biomass) of macronutrients in *Eucalyptus urophylla* at two, three, four, and five years of age, under seedling system and coppice establishment management in Entre Rios, Bahia. Means followed by the same letter do not differ significantly from each other at the 5% probability level according to Tukey’s test. Uppercase letters compare management systems within each age, while lowercase letters compare ages within each management system. (*) Indicates significant interaction between age and management.

**Figure 4:**
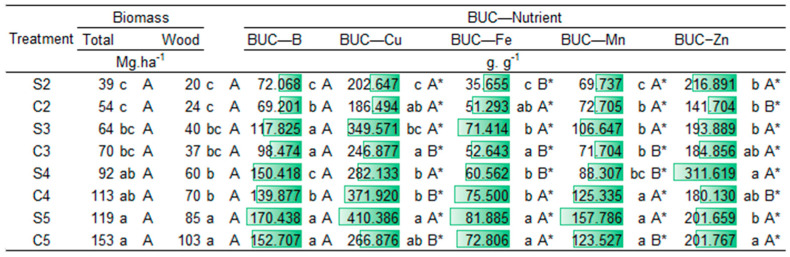
Biological utilization coefficient (BUC—nutrient units per unit of biomass) of micronutrients in *Eucalyptus urophylla* at two, three, four, and five years of age under seedling system and coppice management in Entre Rios, Bahia. Means followed by the same letter do not differ significantly from each other at the 5% probability level according to Tukey’s test. Uppercase letters compare management systems within each age, while lowercase letters compare ages within each management system. (*) Indicates significant interaction between age and management.

Regarding establishment management, potassium and boron showed no differences between the two systems. In the seedling system, there was a higher BUC index for N, P, and at 2 and 4 years for S and Zn. In the coppice, higher BUC values were observed for Mg and, at 2 years, for Ca. Regarding micronutrients Cu, Fe, and Mn, the BUC showed differences between managements according to tree age. Copper and manganese showed higher BUC at 3 and 5 years in the seedling system and at 4 years in the coppice, with no significant difference at age two. On the other hand, iron demonstrated higher utilization efficiency at 2 and 4 years in the coppice and at 3 years in the seedling system, with no significant differences at 5 years (Figure 3 and Figure 4).

Among the nutrients, micronutrients exhibited the highest values of the Biological Use Coefficient (BUC), calculated as the ratio between woody biomass and the total amount of nutrient accumulated in the plant. This trend is expected, as micronutrients are required in smaller quantities by the plant, meaning that even small accumulations support relatively high biomass production. In contrast, macronutrients tend to show lower BUC values because they are demanded in much larger amounts to sustain growth and metabolism. The sequence of average BUC values among treatments, from highest to lowest, was as follows: Zn > Cu > B > Mn > Fe > S > P > Mg > Ca > K > N. This pattern reflects the typical plant nutrient demand and the efficiency of biomass conversion per unit of nutrient.

With increasing age, the biological utilization efficiency of B > Fe > Cu more than doubled when comparing the ages of two and five years. For manganese and zinc, both planting systems had an increase in BUC of 177% and 93%, respectively, between the ages of 2 and 5 years. The seedling system tended to have a higher BUC at almost all ages, except at three years. In the three-year-old treatment, where the seedling and coppicing systems were on more nutritionally similar soils (PCA), coppicing tended to have a higher BUC, except for zinc.

**Figure 5:**
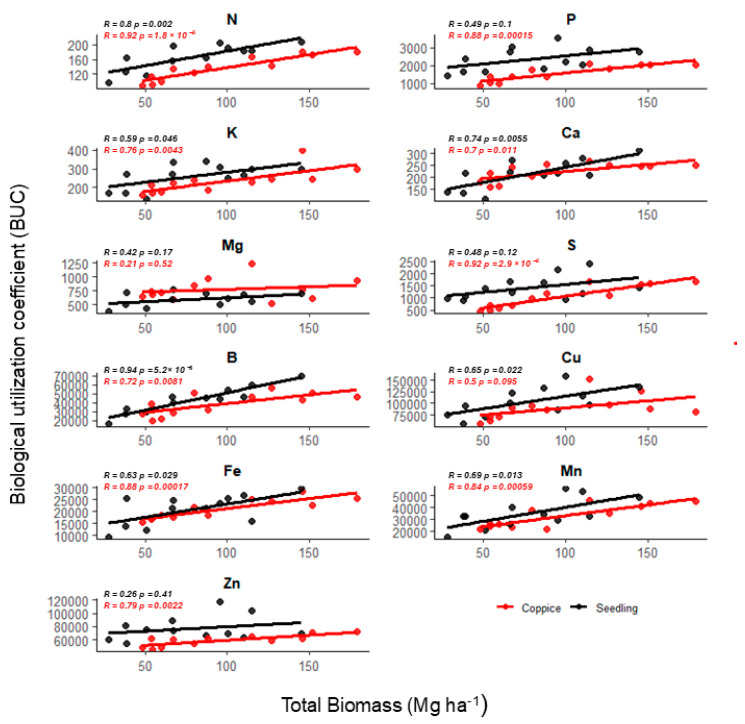
Correlation between the biological utilization coefficient (BUC) and total biomass in *Eucalyptus urophylla* trees aged two to five years under a coppicing and seedling management system in Entre Rios, Bahia, Brazil.

The biological utilization coefficient (BUC) showed different relationships with total biomass for macro and micronutrients in the seedling stage (Figure 5). For macronutrients (N, P, K, Ca, Mg, and S), moderate to strong positive correlations with total biomass were observed, with correlation coefficients (R) ranging from 0.42 to 0.74. The highest R values were found for calcium (Ca) (R = 0.74) and nitrogen (N) (R = 0.68).

Among micronutrients (B, Cu, Fe, Mn, Zn), correlations were generally weaker, with R values between 0.22 and 0.44. Boron (B) and manganese (Mn) presented slightly higher correlations (R = 0.34 and R = 0.43, respectively).

In the coppice stage (Figure 5), BUC correlations with total biomass were generally stronger than in the seedling stage. For macronutrients, the R values were notably higher, ranging from 0.62 to 0.92, with potassium (K) (R = 0.75) and sulfur (S) (R = 0.92) exhibiting the strongest relationships. Micronutrient correlations also increased compared to seedlings, with R values from 0.50 to 0.88. The highest correlations were observed for iron (Fe) (R = 0.88) and manganese (Mn) (R = 0.84).

### 2.3. Carbon Stock

The data obtained on carbon stock in coarse roots reveal significant differences between the seedling and coppice systems. The coppice system exhibited a carbon stock that was 246% higher at 2 years, 139% higher at 3 years, 86% higher at 4 years, and 72% higher at 5 years compared to the seedling system. In the seedling system, the carbon stock increased over the years. The results reveal significant differences across treatments in terms of aboveground biomass, root biomass, and carbon sequestration in the roots. Treatment C5 showed the highest total aboveground biomass with 124.97 Mg ha^−1^, followed closely by treatment S5 with 101.71 Mg ha^−1^. Root biomass was also notably higher in the C5 treatment, with 27.56 Mg ha^−1^, compared to S5’s 16.82 Mg ha^−1^. Additionally, treatments from the coppice group (C2, C3, C4, and C5) exhibited consistently higher carbon storage in the roots, with values ranging from 17.08 to 17.70 Mg ha^−1^, significantly surpassing the seedling treatments. This data underscores the enhanced carbon sequestration potential in the coppice treatments, particularly C5, which combined high aboveground biomass and root carbon storage. These results highlight the effectiveness of the C treatments in promoting both biomass production and carbon sequestration (Table 1).

In contrast, the coppice system showed a relatively constant carbon stock in coarse roots. These data indicate a continuous increase in carbon stock in the coarse roots of the seedling system, while the coppice system maintains higher carbon stock values with little variation over the years.

## 3. Discussion

The total average biomass at five years in the establishment management systems was 152.53 Mg ha^−1^ for the coppice and 118.53 Mg ha^−1^ for the seedling system (Figure 1). An intermediate value was found by Barros [17], who reported a total biomass of 124.93 Mg ha^−1^ for the same *Eucalyptus urophylla* clone at six years of age in a seedling system, located in Vitória da Conquista, Bahia. The soil used in the Barros [17] study was a dystrophic Yellow Latosol, with an annual precipitation of approximately 700 mm.

The influence of age varies among tree compartments due to differences in their physiological functions, growth strategies, and turnover rates. Coarse root biomass, for instance, tends to increase over time because these are permanent structures that continue developing throughout the rotation cycles, especially in systems like coppicing, which reuse the root system from the previous rotation. Therefore, age plays a central role in this compartment. Laclau et al. [18], studying eucalyptus plantations over time, observed that even after the aboveground biomass growth had peaked, the root system continued to expand, with a significant increase in biomass in deep and coarse roots, particularly at older ages. In contrast, compartments such as leaves and fine branches are more dynamic and respond quickly to current environmental conditions, undergoing faster turnover and being less influenced by age. Thus, age has a more significant role in long-lived structural compartments, such as coarse roots and stems, than in those with high turnover rates or growth that is strongly conditioned by other factors.

The trend of greater biomass accumulation in the coppice treatment and the soil characteristics of the treatment area corroborate the findings of Gonçalves et al. [12], which highlight the high volumes of rainfall and well-drained soils in the region as the main reason for the second rotation’s production being equal to or even higher than that of the first rotation.

The greatest accumulation of biomass in coarse roots was observed in the coppice establishment management treatment. This finding is consistent with studies by Barros [17], who assessed *Eucalyptus urophylla* clones at six years of age in the municipality of Vitória da Conquista, Bahia, and reported 18.51 Mg ha^−1^ of coarse root biomass in a dystrophic Yellow Latosol. Similarly, Gonçalves et al. [19] found 20 Mg ha^−1^ of coarse root biomass in a seven-year-old *Eucalyptus grandis* plantation in the municipality of Itatinga, São Paulo, located on a dystrophic Red-Yellow Latosol. Despite these findings, this study highlighted a relatively modest difference, with management being the primary factor influencing the greater growth of coarse roots in the coppice system. Eufrade-Junior et al. [20] further supported these results, noting that the fuel quality of biomass from a short rotation coppice of *Eucalyptus* remained consistent across varying planting densities and fertilization levels, when compared to conventional stand plantations established from seedlings.

In line with these observations, a clear distinction in coarse root biomass was noted between the management systems, with significantly higher values in the coppice system. This can be explained by the presence of a pre-established root network in the coppice system, which allows for faster regrowth and more effective soil exploration, leading to increased biomass accumulation. In contrast, the seedling-based system is still in the early stages of root development, temporarily limiting its biomass accumulation potential during the initial growth phases. Moreover, Silva et al. [21] found that areas managed under coppicing exhibited improved soil permeability, which likely facilitates deeper root penetration and expansion, further contributing to the observed differences in coarse root biomass.

A biomass production survey conducted by Gatto et al. [22] in a stand of a *Eucalyptus urophylla* x *E. grandis* hybrid at five years of age in the Federal District showed a component distribution similar to that found in our study at ages 2 and 3 years: wood > roots > branches > bark > leaves. However, in a study conducted by Barros [17] at 6 years of age in Vitória da Conquista, Bahia, the biomass distribution followed a different order: stem (64.9%), roots (18.8%), branches (10.8%), leaves (2.8%), and bark (2.7%). Regarding the distribution of components found in the literature, there are several citations that differ from our results, especially regarding the branch, bark, and leaf components. However, wood always occupies the first position, except in very young plantations [23].

Our study showed roots as the second component with the highest production at all ages and establishment management systems. Biomass accumulation is directly related to soil and climatic conditions and the local productive capacity of the plantation area [17,24,25,26,27]. This study is located in a region with good rainfall indices (between 1100 mm and 1300 mm), compared to some regions of Brazil, but it still experiences periods of water deficit throughout the year. According to Gonçalves [28], there is an increase in root biomass production in areas experiencing more pronounced conditions of nutritional or water stress. This leads trees to accumulate more assimilates in the roots [11]. Intact stump percentage significantly correlated with both tree uniformity (PV50) and mean annual increment (MAI), reflecting higher productivity and uniformity, which were further influenced by light use efficiency and treatment light capture [29].

Regarding the data on component behavior over time, they are justified according to the description provided by Schumacher et al. [30], where during the initial growth phase of plants, most of the assimilates synthesized by the plant are directed towards crown and root system formation. After canopy closure, trees accumulate more biomass and nutrients more intensively in the stem, as at this stage the crowns are in a phase of relative stability due to self-shading. Younger trees with smaller diameters (first year) show greater biomass accumulation in the crown compared to the trunk wood, a behavior that decreases as diameters increase [31].

The biological utilization coefficient (BUC) describes the conversion rate between the quantity of each nutrient per unit of biomass, allowing an understanding of the efficiency in nutrient utilization in each system for wood biomass production. Therefore, the more efficient the conversion, the higher the value of the BUC [32].

The increase in BUC with age highlights the possible relationship between nutrient utilization efficiency and tree development over time, mainly due to nutrient cycling processes known as biochemical and biogeochemical processes. These processes occur through forest stability after canopy closure, with nutrient translocation by the plant and its relationship both internally and with the soil [16,33].

This may not necessarily indicate high productivity [9,13,33,34]. Although these indices do not provide a complete and definitive view, when used correctly, they can be a useful tool for understanding the processes responsible for tree growth [33] and assisting in fertilizer recommendations. The highest BUC values were observed in the seedling system, suggesting greater efficiency in converting nutrients into wood dry matter. However, in the coppice, although the accumulation of some nutrients was relatively higher, biomass production was similar, with a slightly higher tendency for the coppice establishment treatment.

The higher BUC values for magnesium (Mg) in coppice systems are of particular interest. Magnesium plays a crucial role in photosynthesis as the central atom of chlorophyll, as well as in stabilizing the root system and supporting overall plant growth. The increased efficiency of magnesium uptake observed in the coppice system suggests that this system may exhibit a more developed and efficient nutrient cycling process. According to Dias et al. [35], their study demonstrated that the coppice management system, with its established root network, promotes better nutrient recycling, resulting in higher nutrient use efficiency, especially for magnesium. This enhanced efficiency could potentially reduce the need for frequent fertilization, as the system’s ability to recycle nutrients from previous cycles allows for a more sustainable and cost-effective approach to nutrient management. The authors also observed that the coppice system’s rapid regeneration and deeper root penetration may contribute to this improved nutrient cycling, further supporting the idea that it may be more resource-efficient than seedling-based systems.

Potassium (K) and Boron (B) did not show significant differences between the coppice and seedling systems. This result can be explained by the high mobility of these nutrients in both soil and plant systems, as highlighted by Andrade et al. [36]. Both potassium and boron are efficiently translocated within plants, allowing them to move easily through the soil profile and be absorbed by roots regardless of the management system. This high mobility ensures that these nutrients are readily available to the plant, even in the presence of variations in root depth between the coppice and seedling systems. Furthermore, the availability of K and B is less influenced by soil depth or root distribution, which may account for the lack of differentiation between the systems observed in this study. Therefore, the similar accumulation of these nutrients in both management systems suggests that their uptake is not strongly dependent on the root development pattern or system management type.

The higher BUC values for N, P, and Mg in the seedling system, as well as in some cases for S, Cu, and Zn, may indicate nutritional restrictions. This may be due to water restriction, due to the low permeability of cohesive soils, which can also prevent or hinder the transport of nutrients to the roots, leading to an increase in BUC values.

Among the nutrients, micronutrients showed the best conversion rate. These nutrients are of utmost importance for plant growth and are absorbed in smaller quantities, resulting in lower accumulation and higher efficiency per unit of wood biomass. Similar trends have been reported by Laclau et al. [16], who observed high BUC values for micronutrients in fast-growing *Eucalyptus* plantations, and by Rocha et al. [37], who emphasized the role of nutrient use efficiency in biomass accumulation under different site conditions and genetic materials. These findings highlight the importance of understanding nutrient dynamics not only in terms of absolute accumulation but also in relation to biomass productivity.

The BUC increased its correlation strength with total biomass from the seedling to coppice stages, suggesting that nutrient utilization efficiency becomes more synchronized with biomass accumulation as the stand matures. Similar trends have been observed in other studies where nutrient use efficiency is optimized in later developmental stages of *Eucalyptus* spp. [38,39].

In seedlings, the moderate correlations for macronutrients may reflect the early establishment phase, where nutrient uptake and biomass production are not fully coordinated. In particular, the lower correlations for micronutrients could indicate that their roles are less directly tied to biomass accumulation during initial growth, as previously described by Laclau et al. [40]. In the coppice system, the high correlation values, especially for sulfur (S), potassium (K), and iron (Fe), highlight a more efficient physiological adjustment of nutrient use post-harvest. Coppice systems often benefit from a developed root system, leading to improved nutrient foraging and remobilization from the soil and residual biomass [41,42].

The particularly strong correlation observed for sulfur in the coppice phase (R = 0.92) suggests its critical role during the regrowth period, likely due to its involvement in protein synthesis and metabolic activation under high growth demand [43]. The enhanced micronutrient use, especially iron and manganese, aligns with the necessity for efficient enzymatic activities supporting vigorous regrowth in coppiced stands [44]. Thus, our findings reinforce that coppicing can enhance nutrient utilization dynamics, potentially contributing to sustainable productivity if nutrient availability is maintained.

These findings emphasize the importance of understanding nutrient use efficiency in varying silvicultural contexts. Differences in nutrient utilization efficiency, besides being related to the capacity for absorption, translocation, and conversion of nutrients into biomass of each genotype, are also influenced by genotype–environment interactions [45]. Thus, nutrient utilization efficiency allows for the identification of management practices, such as fertilization, that contribute to forest sustainability. Knowing the efficiency in using a nutrient and the corresponding biomass production expectation, it becomes possible to estimate the amount of nutrients needed for an adequate nutritional balance in the next rotation cycle [46]. These insights can guide policy-making by providing evidence-based recommendations for resource allocation, enabling more targeted and cost-effective fertilization strategies. Furthermore, they support practical forestry decisions by helping forest managers optimize nutrient management practices to enhance productivity while minimizing environmental impacts.

Carbon sequestration plays a critical role in mitigating climate change. The carbon stocks in forest ecosystems, especially in the form of coarse roots, contribute to long-term carbon storage, which is essential for the global carbon cycle.

The difference in carbon accumulation in coarse roots between the seedling and coppice systems can be attributed to several factors, including the growth dynamics and resource allocation strategies of each system. Coppicing, which involves the regrowth of trees after harvesting, often leads to a significant increase in carbon storage in the roots due to the perennial nature of the root system. Studies show that coarse roots of plants in coppice systems can represent up to 60% of the total belowground biomass, serving as an important carbon reservoir [16]. This dynamic is supported by research indicating that in eucalyptus coppices, regrowth contributes to a considerable carbon stock, which is essential for climate change mitigation [47].

The seedling system tends to focus on the vertical growth of trees, resulting in a progressive accumulation of carbon in the roots over time. Although the carbon stock in coarse roots increases as the trees mature, the growth rate may be less pronounced compared to the coppice system. The maintenance of carbon stock in the roots within the coppice system, with relatively constant values, reflects the efficiency of regrowth in conserving root biomass during harvesting cycles [4]. A study conducted by O’Brien et al. [48] found that management systems utilizing coppicing tend to have a more stable carbon accumulation, which can be crucial in scenarios of climate change.

The benefits associated with the higher accumulation of carbon in coarse roots in coppice systems include an increased capacity for carbon sequestration in both the short and long term, which is essential for climate change mitigation. Additionally, the presence of a significant root mass can improve soil structure and fertility, promoting a healthier environment for plant growth [49]. For example, research by O’Connell et al. [50] demonstrates that root diversity in coppice systems can enhance soil stability, which is vital for the overall health of the ecosystem.

The age of the trees in both systems also plays a crucial role in the observed differences. While the seedling system shows a continuous increase in carbon stock in the roots over the years, the coppice system maintains a more stable level, reflecting the inherent growth dynamics of each system. This stability in the coppice system can be interpreted as a sign of a well-adjusted system, where regrowth maximizes carbon use efficiency without relying solely on vertical growth [51]. According to Van der Werf et al. [52], this approach not only ensures more consistent carbon sequestration but also provides resilience in the face of environmental stresses.

## 4. Materials and Methods

### 4.1. Study Area

**Figure 6:**
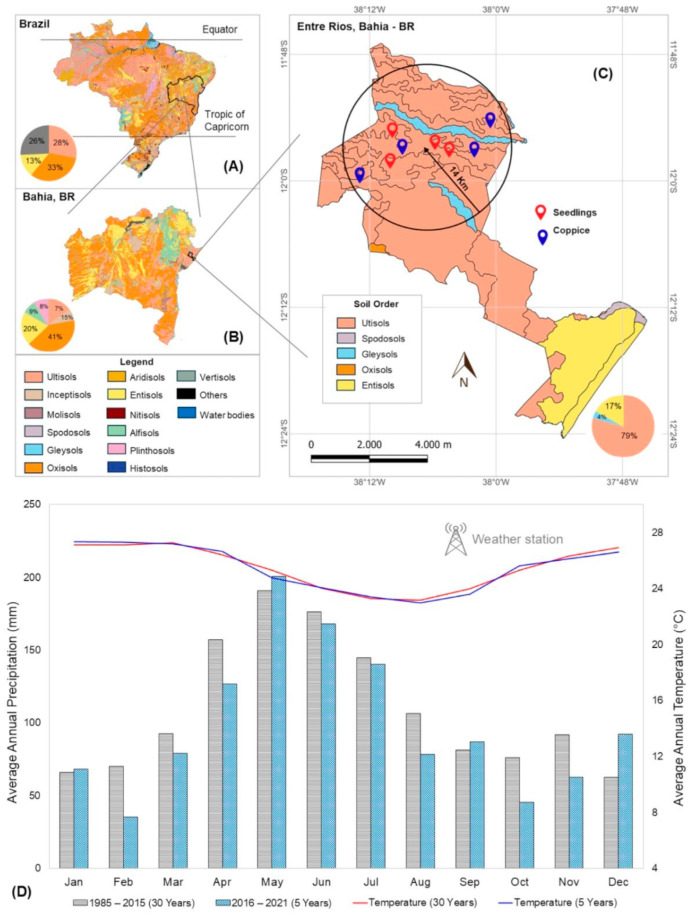
Location map (**A**): country, (**B**): state, and (**C**): city with soil orders, according to IBGE data. Data on the annual distribution of rainfall and monthly average temperature (**D**) from the automatic weather station (WS-GP2 Automatic Weather Station model), with a 30-year historical record (Xavier) prior to the start of the installation of the study area and at 5 years when the study was conducted, in seedling system (S) and coppice (C) stands distributed in the municipality of Entre Rios, Bahia, Brazil.

The *Eucalyptus urophylla* stands are located in the northern coastal region of the state of Bahia, in the municipality of Entre Rios, positioned between the geographical coordinates of 11°51′08″ W latitude south and 38°06′52″ S longitude west. The soils in this region are predominantly Ultisols (Figure 6), with a texture ranging from sandy to medium, characterized as dystrophic (Table 2), and with a slightly undulating relief.

The region’s climate was monitored by a meteorological station considering an influence radius of 14 km. Therefore, the studied stands are part of the same climatic zone, with an average annual temperature between 25 and 26 °C, characterized as a humid tropical climate (Af) according to the Köppen classification, with rainy winters and dry summers. The average annual precipitation is 1200 mm [53].

A multivariate principal component analysis (PCA) was used to characterize the study areas, utilizing the chemical and physical attributes of the soil. PCA is a mathematical formula used to reduce data dimensionality, aiming to identify patterns in the study areas based on the attributes evaluated in the soil analysis, objectively expressing their similarities and highlighting their differences.

The principal component analysis consisted of two components (dim. 1 and dim. 2) at two different depths in the soil profile. Regarding the first 20 cm depth, dim 1 explains 73.7% of the data, while the second explains 12%, accounting for a total of 85.7% of the data variation. The variables that contributed most to explaining dim 1 were: sand, clay, P, K, OM, Ca, Mg, and B, and they also contributed to the formation of dim 2. At the 20 to 40 cm depth, dim 1 explains 66.6% of the data, while the second explains 16.5%, accounting for a total of 83.1% of the data variation. As with the previous depth, similar contributions from the same variables were obtained to explain dimensions 1 and 2. Dim 1 separated the studied treatments by differences in soil attributes (Table 2). The clustering of the areas in this study is mainly due to the soil texture-related attributes (clay and sand), which have the largest vectors, with a clear negative correlation (Supplementary Materials).

The results obtained from the PCA offer a valuable starting point for understanding how the particularities of each area’s soil attributes influence the data discussed in this work. Even though the studied areas were selected in locations with the same characteristics, such as average annual precipitation range, soil order, and suborder, PCA revealed a separation of treatments into two groups. This indicates that soil attributes play a significant role in the observed differences between the analyzed treatments, highlighting the importance of considering these variations in the discussion of this work.

### 4.2. Experimental Design and Treatments

The experiment was analyzed in a completely randomized design (CRD), consisting of 8 treatments and 4 replications. Each experimental plot consisted of 36 plants, distributed in 6 rows of 6 plants each, with a spacing of 9 m^2^ per individual, totaling 576 plants per treatment (with 144 plants per plot).

The eight treatments included ages of 2, 3, 4, and 5 years post-planting under seedling system management (S2, S3, S4, and S5), and post-harvest under coppice management (C2, C3, C4, and C5). The selection of these ages aimed to track the different nutritional stages of each stand. The species used in the study was a clone of *Eucalyptus urophylla*, selected for its adaptability to the region.

In the site preparation, dolomitic limestone was applied in all treatments. In the seedling system, fertilization was carried out in three distinct stages: a basal fertilization during subsoiling at a depth of 20 to 30 cm, a topdressing (surface) fertilization, and a maintenance fertilization, both applied mechanically in a continuous strip (Table 3). Meanwhile, in the coppice, fertilization occurred in a single application, as topdressing. Some treatments received supplementary fertilization as needed when reported by the field team. Regarding the dates and periods of fertilization, they varied according to the rainfall window and operational planning. The coppicing management was conducted early, around six months post-harvest, promoting a single sprout, except near planting gaps, where two stems were maintained to compensate.

### 4.3. Aerial Biomass

Each treatment was represented by an experimental unit consisting of a permanent plot, which was subdivided into ten diameter classes based on the frequency distribution of the diameter at breast height (DBH) of the inventoried trees. From this classification, the three central diameter classes (Classes 5, 6, and 7) were selected for nutrient sampling, as they represented intermediate individuals and were therefore considered more representative of the population.

The selected trees were felled and immediately fractionated in the field into the following compartments: stem (wood + bark), leaves, and branches. Each compartment was separated and weighed entirely using a portable precision balance to determine the total fresh biomass per compartment. Representative samples of each compartment were collected, placed in pre-identified paper bags, weighed in the field, and subsequently transported to the laboratory, where they were oven-dried at 65 °C with forced air circulation until reaching constant weight.

The dry biomass of each compartment was calculated using the ratio between the fresh and dry mass of the samples. The dry mass of the stem compartment (wood and bark) was obtained through composite samples formed by disks collected from different positions along the trunk (base, middle, and top), following a destructive sampling protocol.

After drying, all samples were ground using a Wiley-type mill with a 1 mm mesh sieve, homogenized, and subjected to chemical analysis in the laboratory. Nutrient contents were determined according to the methodology described by Malavolta et al. [54], allowing the quantification of average concentrations of macronutrients (g kg^−1^) and micronutrients (mg kg^−1^) for each plant compartment and age class evaluated.

### 4.4. Root System Biomass

The belowground biomass assessment was conducted through direct sampling of the stump and coarse roots (defined as roots with a diameter greater than 2 mm), but only in forest stands aged three and five years, under both coppice and planted seedling systems. In each treatment, four trees were selected for destructive root sampling, totaling eight trees per forest management system. Manual excavation was carried out around the selected trees, with the assistance of a manual crane with an 8-ton lifting capacity, which enabled the complete extraction of the stump-root system while minimizing biomass loss.

After extraction, coarse roots were manually cleaned of adhering soil, separated from the stump, and weighed in the field using a portable precision scale to determine total fresh biomass. Representative subsamples were collected from each root system, placed in pre-identified paper bags, weighed fresh, and transported to the laboratory. Samples were oven-dried at 65 °C in a forced-air circulation oven until reaching constant weight. The total dry biomass of each sample was estimated by applying the fresh-to-dry mass ratio.

Once dried, all subsamples were ground using a Wiley type knife mill equipped with a 1 mm mesh sieve, homogenized, and submitted to chemical analysis. Nutrient concentrations in the coarse root biomass were determined following the procedures described by Tedesco et al. [55], allowing quantification of macronutrients (g kg^−1^) and micronutrients (mg kg^−1^) for each treatment and age class.

To estimate root system biomass for all age classes, allometric equations were developed separately for the *Eucalyptus urophylla* stands under the seedling and coppice systems, using total stem height (S) as the independent variable, according to the Schumacher and Hall [56] model. These equations were derived from trees destructively sampled at three and five years of age. For the remaining age classes, root biomass was estimated indirectly using the height-based equations, selecting the model with the highest coefficient of determination (R^2^) for each system to ensure the most accurate prediction. Root biomass was then estimated using the following equation:

Y = β0 + β1 × (h)(1)

In which:

Y = biomass, in Kg
β0 and β1 = coefficients of the models.
h = height of the individuals.

Equation for seedling system:

Y = 1.0885 × (h) – 9.4496(2)

Equation for coppice:

Y = 0.9519 × (h) + 3.8895(3)

For the calculation of the biomass expansion factor (BEF) of the trees, the formula indicated by the IPCC [57] was used:

BEF = (Wcanopy + Wtunk)/Wtunk = Waboveground/Wtunk(4)

where: BEF = biomass expansion factor (dimensionless); Wcanopy = dry weight of the tree canopy (g); Wtunk = dry weight of the tree trunk (g); Waboveground = dry weight of the tree trunk + dry weight of the tree canopy (g).

The root-to-shoot ratio was obtained using Formula (2), according to the IPCC [57]:

R = Wroot/Waboveground(5)

where: R = root-to-shoot ratio (dimensionless); Wroot = dry weight of the tree roots (g); Waboveground = dry weight of the aboveground part of the tree (g).

Finally, the carbon stock in the roots was calculated using the formula adapted from Corte et al. [58] (3).

R = Wroot × BEF × (1 + R) × CF(6)

where: CF is a conversion factor equivalent to 0.47, as suggested by the IPCC [57] for the genus *Eucalyptus*.

### 4.5. Biological Utilization Coefficient

The biological utilization coefficient (BUC) is an index that reflects the efficiency with which a plant converts a given quantity of a nutrient into biomass, particularly wood biomass. In other words, it indicates how many kilograms of wood are produced per unit of a specific nutrient absorbed and retained in the plant tissues. This metric is useful for evaluating nutrient-use efficiency among different species, genotypes, or management systems.

In this study, BUC was calculated as the ratio between the dry biomass of wood and the total amount of a given nutrient contained in the whole plant (sum of stem, bark, branches, leaves, and roots), with both variables expressed in the same units. The calculation follows the methodology described by Barros et al. [59] and Bazani [33], and is illustrated by the following formula:

BUC = Amount of Biomass in Wood/Amount of Nutrient in the Plant(7)

### 4.6. Data Analysis

The data from biomass compartments, nutrient content, and concentration, as well as the biological utilization coefficient, underwent descriptive statistical analyses, followed by tests for normality and homogeneity of the data. Additionally, an analysis of variance (ANOVA) was conducted in a factorial design considering the factors of age and management to identify significant effects. The design used was completely randomized, with trees selected as sampling units. When necessary, means were discriminated using Tukey’s test, with a significance level of 5%, using the statistical software R^®^ version 4.0.3 (R Development Core Team, 2020). The estimation of root biomass was based on the joint analysis of the coefficient of determination (R^2^), standard error of the estimate (Sy.x), and graphical analysis of percentage residuals.

## 5. Conclusions

This study demonstrated that both coppice and seedling planting systems in Ultisol soils under a humid tropical climate resulted in similar biomass production patterns, with the greatest biomass accumulation observed in wood as the plantations aged.

Although the seedling system showed higher biological utilization coefficients (BUC) for nitrogen (N) and phosphorus (P), the coppice system stood out for its greater efficiency in the use of magnesium (Mg). More importantly, even in soils with lower nutrient content and reduced fertilization, the coppice system achieved nutrient utilization efficiency comparable to the seedling system.

These results suggest that, to promote nutritional efficiency and carbon storage, especially in nutrient-poor soils, tailored fertilization strategies may be key to optimizing the coppice system’s performance. The stability of carbon stocks in the coarse roots of the coppice system provides an advantage in terms of carbon sequestration, improving soil structure and fertility, and consequently, the sustainability of the forest ecosystem.

In this study, the coppice system showed a higher carbon accumulation in coarse roots (17.23 Mg ha^−1^) compared to planting with seedlings (9.99 Mg ha^−1^), representing a 72.4% increase. This highlights the importance of accounting for management practices when estimating belowground carbon stocks. Commonly used parameters such as the biomass expansion factor (BEF) and root-to-shoot ratio (R) are typically based on reference values for eucalyptus plantations without considering the influence of different management systems.

Therefore, studies like this contribute to refining these estimates and provide a basis for more representative evaluations of the coppice system. For future applications, strategies that optimize nutrient use in low-fertility soils and enhance fertilization efficiency should be integrated with practices that retain harvest residues in the field, as these residues play a key role in nutrient cycling and carbon maintenance.

## Figures and Tables

**Table 1:** Carbon stock in roots (Mg ha^−1^), biomass expansion factor (BEF), root ratio (R) and biomass (W—of stem, shoots and roots) of *E. urophylla* under seedling and coppice management.

Treatment	BEF	R	W_tunk_	W_aboveground_	W_root_	Carbon in the Roots		
			kg			Mg ha^−1^		
S2	1.37	0.20	23,629	32,475	6588	5.12	c	B
S3	1.18	0.20	45,340	53,716	10,760	7.19	b	B
S4	1.14	0.19	68,491	77,776	14,461	9.15	a	B
S5	1.08	0.17	93,780	101,714	16,820	9.99	a	B
C2	1.27	0.55	27,520	35,072	19,120	17.70	a	A
C3	1.16	0.45	41,678	48,209	21,785	17.20	a	A
C4	1.10	0.29	79,601	87,948	25,498	17.08	a	A
C5	1.09	0.22	114,701	124,972	27,560	17.23	a	A

Means followed by at least one same letter, uppercase for management and lowercase for age, do not differ from each other at the 5% probability level, according to Tukey’s test.

**Table 2:** Chemical and granulometric characterization of soil at depths of 0–20 cm and 20–40 cm in *Eucalyptus urophylla* planting sites at 2, 3, 4, and 5 years under seedling system and coppice management, in Ultisols and humid tropical climate.

Treatment	Depth	Coarse Sand	Thin Sand	Silt	Clay	M.O	pH	Al	Ca	Mg	K	Al + H	SB	V	m	P	B	Cu	Fe	Mn	Zn
	(cm)	g kg^−1^				g dm^−3^		mmol_c_ dm^−3^						%		mg dm^−3^					
S2	0–20	420	194	111	275	23	5.0	0.23	26	3.5	0.68	28	30	52	0.7	8	0.41	1.0	40	1.67	0.47
	20–40	421	184	111	283	18	4.9	0.85	21	3.2	0.54	31	24	43	3.8	6	0.47	1.0	31	0.97	0.27
C2	0–20	445	244	95	217	20	4.9	1.38	12	4.1	0.67	35	17	33	7.2	9	0.50	0.7	35	0.47	0.53
	20–40	435	211	104	250	16	4.6	4.07	8	3.9	0.39	44	13	22	23.8	5	0.47	0.9	21	0.20	0.27
S3	0–20	483	276	74	167	15	5.0	0.98	10	2.9	0.55	32	13	29	6.9	6	0.44	0.7	21	0.53	0.27
	20–40	459	210	97	233	14	4.8	2.03	11	4.6	0.63	44	16	27	11.6	5	0.36	0.8	18	0.27	0.17
C3	0–20	522	273	71	133	13	4.9	0.98	12	3.5	0.53	41	16	28	6.4	5	0.27	0.7	31	0.93	0.40
	20–40	516	264	70	150	8	4.8	1.61	9	3.7	0.41	41	13	24	9.9	4	0.31	0.8	27	0.37	0.17
S4	0–20	344	205	126	325	20	5.1	0.63	22	5.6	1.07	30	29	49	2.8	21	0.71	1.2	38	1.17	0.80
	20–40	347	192	120	342	13	4.8	2.53	16	4.0	0.78	38	21	35	13.0	11	0.67	1.0	24	0.50	0.37
C4	0–20	448	237	98	217	17	5.2	0.00	20	6.2	0.48	24	27	52	0.0	8	0.34	0.9	21	1.13	0.33
	20–40	427	218	105	250	11	4.8	2.33	9	3.7	0.47	46	13	22	15.1	7	0.46	0.7	14	0.30	0.13
S5	0–20	338	179	141	342	23	4.9	0.69	25	6.5	1.60	34	33	49	2.2	16	0.49	1.5	38	0.83	0.60
	20–40	364	122	139	375	18	4.8	1.81	15	4.5	1.29	37	21	36	8.1	11	0.64	1.1	24	0.67	0.23
C5	0–20	445	238	92	225	17	4.9	0.92	21	5.0	0.65	32	27	45	3.6	10	0.33	0.7	24	0.60	0.33
	20–40	415	180	114	292	16	4.9	1.87	14	4.4	0.50	33	19	36	12.1	6	0.54	0.8	18	0.30	0.17
Average	0–20	431	231	101	238	18	5.0	0.73	18	4.7	0.78	32	24	42	3.7	10	0.44	0.9	31	0.92	0.47
	20–40	423	198	108	272	14	4.8	2.14	13	4.0	0.63	39	17	31	12.2	7	0.49	0.9	22	0.45	0.22
General Average	0–40	427	214	104	255	16	4.9	1.43	16	4.3	0.70	36	21	36	8.0	9	0.46	0.9	26	0.68	0.34
Standard Deviation	0–40	55	17	21	68	4.0	0.1	1.0	5.8	1.0	0.3	6.1	6.6	10.3	6.0	4.2	0.1	0.2	7.8	0.4	0.2
CV%	0–40	13%	8%	20%	27%	24%	3%	69%	37%	23%	47%	17%	32%	28%	75%	49%	27%	24%	30%	58%	51%

Where: Organic Matter (OM), pH (water); N by the Kjeldahl method; extractable P and K by Mehlich^−1^; exchangeable Ca, Mg, and Al by KCl 1 mol L^−1^. H + Al = potential acidity, SB = sum of bases, V = base saturation, m = aluminum saturation, CV = coefficient of variation. Composite samples consisting of 10 individual samples collected from the experimental area were used. Treatment: S2—Seedling system at 2 years post-planting; C2—Coppice at 2 years post-harvest; S3—Seedling system at 3 years post-planting; C3—Coppice at 3 years post-harvest; S4—Seedling system at 4 years post-planting; C4—Coppice at 4 years post-harvest; S5—Seedling system at 5 years post-planting; and C5—Coppice at 5 years post-harvest.

**Table 3:** Fertilization applied in the experimental area.

Treatment	Silvicultural Operations	Fertilizer Formulation	Quantity (Kg)
S2	Liming	Dolomitic limestone	1500
	base fertilization	NPK 06-30-06 + Micro	250
	Topdressing fertilization	NPK 10-00-30 + Micro	250
	Maintenance fertilization	NPK 09-00-30 + 0.75 B	300
C2	Liming	Dolomitic limestone	1500
	Topdressing fertilization	NPK 10-08-22 + Micro	600
S3	Liming	Dolomitic limestone	2000
	base fertilization	NPK 06-30-06 + Micro	200
	Topdressing fertilization	NPK 10-08-22 + Micro	250
	Maintenance fertilization	NPK 10-00-30 + Micro	400
C3	Liming	Dolomitic limestone	1000
	Topdressing fertilization	NPK 10-08-22 + Micro	600
	Additional fertilization	coated urea + 0.5% B	150
S4	Liming	Dolomitic limestone	1500
	base fertilization	NPK 06-30-06 + Micro	200
	Topdressing fertilization	NPK 10-00-30 + Micro	200
	Maintenance fertilization	NPK 10-00-30 + Micro	350
	Additional fertilization	reactive natural phosphate	300
	Booster fertilization	NPK 06-30-06 + Micro	30
C4	Liming	Dolomitic limestone	1500
	Topdressing fertilization	NPK 08-12-25 + Micro	600
S5	Liming	Dolomitic limestone	1500
	base fertilization	NPK 06-30-06 + Micro	200
	Topdressing fertilization	NPK 10-00-30 + Micro	200
	Maintenance fertilization	NPK 10-00-30 + Micro	300
C5	Liming	Dolomitic limestone	1500
	Topdressing fertilization	NPK 10-08-22 + Micro	600

## Data Availability

Datasets are available on request to the authors.

## Supplementary Materials

The following supporting information can be downloaded at: https://www.mdpi.com/article/10.3390/plants14091382/s1, Figure S1: Principal component analysis (PCA) of soil attributes (sand, clay, OM, K, P, Ca, Mg, and B) at depths of: (A) 0–20 cm and (B) 20–40 cm in *Eucalyptus urophylla* forests, at two, three, four, and five years of age, under seedling system and coppice establishment management in Ultisols and humid tropical climate.
