# Supplementary material for: Full-Tree Biomass, Root Carbon Stock, and Nutrient Use Efficiency Across Ages in Eucalyptus Stands Under Seedling and Coppice Systems
Source: Plants (Basel). 2025 May 3;14(9):1382. doi: 10.3390/plants14091382 (PMC12073641; doi:10.3390/plants14091382)
Supplement: Supplementary file 1 [file plants-14-01382-s001.zip › plants-3577606-supplementary.pdf]

Supplementary material for the manuscript **"Full-tree biomass, root carbon stock, and nutrient use efficiency across ages in *Eucalyptus* stands under seedling and coppice systems"**

Supplementary Figure S1

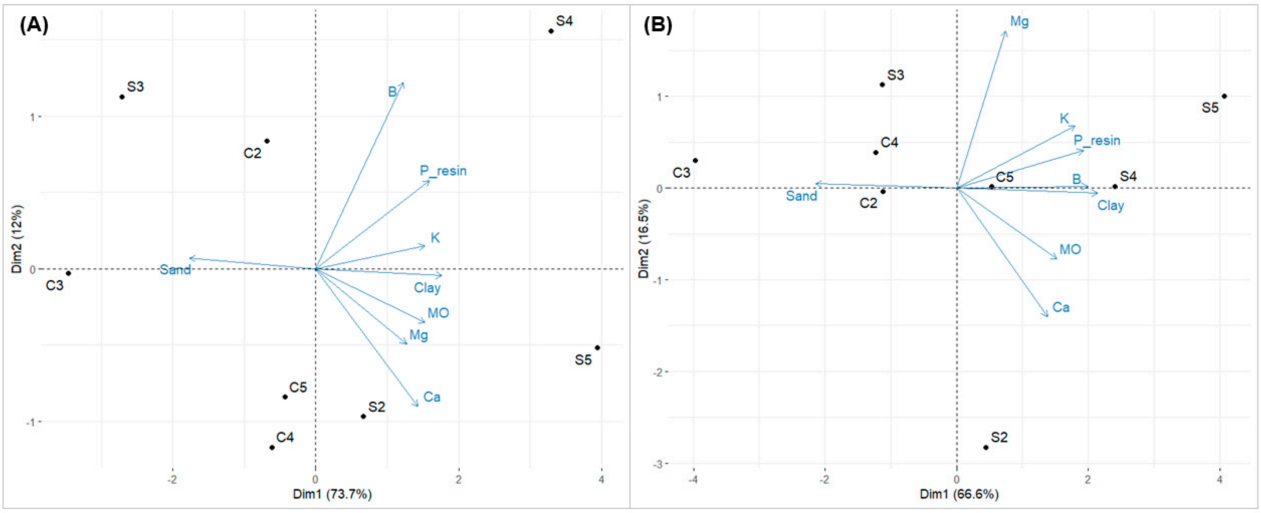

Figure S1. Principal Component Analysis (PCA) of soil attributes (Sand, Clay, OM, K, P, Ca, Mg, and B) at depths of: (A) 0-20 cm and (B) 20-40 cm in *Eucalyptus urophylla* forests, at two, three, four, and five years of age, under seedling system and coppice establishment management in Ultisols and humid tropical climate.
